# Relationship of Emotional Functioning in 9–12-Year-Old Schoolchildren with Key Lifestyles: Sleep Quality and Daily Physical Activity

**DOI:** 10.3390/children13030419

**Published:** 2026-03-19

**Authors:** María del Carmen Carcelén-Fraile, Agustín Aibar-Almazán, Fidel Hita-Contreras, Yolanda Castellote-Caballero

**Affiliations:** 1 Department of Educational Sciences, Faculty of Social Sciences, University of Atlántico Medio, 35017 Las Palmas de Gran Canaria, Spain; carmen.carcelen@pdi.atlanticomedio.es; 2 International Scientific Association on Innovation in Education and Health (ACIINES), 23007 Jaén, Spain; 3 International Network of Educational Law (RIIDE), 35017 Las Palmas de Gran Canaria, Spain; 4 Department of Health Sciences, Faculty of Health Sciences, University of Jaén, 23071 Jaén, Spainmycastel@ujaen.es (Y.C.-C.)

**Keywords:** child mental health, socioemotional development, sleep problems, movement behaviors, primary education

## Abstract

**Highlights:**

**What are the main findings?**

• Sleep problems were the strongest independent predictor of emotional regulation and emotional lability in elementary school children.
• Habitual physical activity was associated with lower emotional instability, but its predictive strength decreased after adjusting for sleep and sociodemographic factors.

**What are the implications of the main findings?**

• Interventions targeting children’s emotional regulation may benefit from prioritizing sleep hygiene as a core component of mental health promotion.
• Promoting physical activity alongside sleep health within a 24 h movement behavior framework may enhance socioemotional development in school settings.

**Abstract:**

Background/Objectives: Sleep problems and physical activity are key lifestyle behaviors associated with children’s socioemotional development. However, their relative contribution to emotional regulation and emotional instability during middle childhood remains insufficiently clarified. This study aimed to examine the associations between sleep problems, habitual physical activity, and emotional regulation in elementary school children, while controlling for sociodemographic and lifestyle confounding variables. Methods: A cross-sectional study was conducted with 386 elementary school children (mean age = 11.15 ± 0.66 years; 45.6% boys) from southern Spain. Emotional regulation was assessed using the Emotion Regulation Checklist (ERC), sleep problems were measured with the Children’s Sleep Habits Questionnaire (CSHQ), and habitual physical activity was evaluated using the Physical Activity Questionnaire for Children (PAQ-C). Age, sex, socioeconomic status, and daily screen time were included as covariates. Pearson correlations and multiple linear regression analyses were performed. Results: Sleep problems were strongly associated with lower adaptive emotional regulation and higher emotional lability. In adjusted regression models, sleep problems emerged as the most robust independent predictor of both emotional regulation and lability. Although habitual physical activity was significantly associated with emotional outcomes at the bivariate level, its predictive strength decreased after adjustment for covariates and did not independently predict adaptive emotional regulation. Conclusions: Sleep problems appear to play a central role in children’s emotional functioning during middle childhood. Interventions aimed at promoting socioemotional well-being may benefit from prioritizing sleep health alongside physical activity within a comprehensive 24 h movement behavior framework.

## 1. Introduction

Childhood represents a critical developmental period characterized by rapid biological, cognitive, and socioemotional changes [1]. During the elementary school years, children acquire essential skills related to self-regulation [2], social interaction [3], academic engagement, and behavioral control [4]. This stage is marked by increased autonomy and expanding social environments, which place growing demands on children’s emotional and cognitive capacities [5]. The establishment of healthy lifestyle habits during this period, including adequate sleep, regular physical activity, and balanced daily routines, plays a decisive role in shaping long-term mental and physical health trajectories [6]. Understanding the factors that influence emotional development during middle childhood is therefore of considerable relevance for both research and preventive interventions [7].

Sleep is a fundamental biological process that plays a crucial role in children’s physical growth, brain maturation, cognitive functioning, and emotional development [8]. During middle childhood, adequate sleep is essential for optimal neurodevelopment, particularly in brain regions involved in executive functioning, emotional processing, and behavioral control [9]. Sleep contributes to memory consolidation, synaptic plasticity, and the regulation of affective responses, processes that are especially active during school-age years [10,11]. Conversely, sleep disturbances or insufficient sleep have been consistently associated with emotional instability, increased irritability, attention difficulties, and behavioral problems [12,13]. Given that sleep patterns undergo significant developmental changes during childhood, including modifications in sleep duration, sleep architecture, and circadian regulation, understanding how sleep quality influences emotional functioning during this period is of particular importance [14].

Emotional regulation refers to the ability to monitor, evaluate, and modify emotional reactions in order to achieve adaptive functioning within social and environmental contexts [15]. In elementary school children, emotional regulation becomes increasingly sophisticated due to progressive maturation of executive functions such as inhibitory control, cognitive flexibility, and working memory [16]. Neurodevelopmental research has shown that prefrontal cortical regions, which support top-down regulatory processes, continue to mature throughout childhood, enabling children to better manage emotional responses and reduce emotional lability [17]. However, this developmental process is highly sensitive to environmental and lifestyle factors. Poor sleep quality may disrupt prefrontal functioning, thereby impairing children’s ability to regulate emotional responses effectively [18]. Indeed, previous studies have shown that sleep problems are associated with increased emotional reactivity, mood dysregulation, and externalizing and internalizing symptoms in school-aged children [19,20].

Physical activity is another behavioral factor closely linked to both sleep and emotional functioning [21]. Regular physical activity during childhood has been associated with improvements in mood, reductions in anxiety and depressive symptoms, enhanced executive functioning, and better self-regulatory skills [22]. Physiological mechanisms such as increased endorphin release, improved stress regulation, and enhanced sleep efficiency may partly explain these benefits [23]. Moreover, physically active children often display more stable sleep patterns and improved sleep quality, suggesting a bidirectional relationship between movement behaviors and sleep regulation [24]. Conversely, insufficient physical activity and high levels of sedentary behavior have been associated with poorer psychological well-being and reduced emotional control [25]. Therefore, physical activity may not only independently influence emotional regulation but also interact with sleep quality in shaping children’s emotional development [26].

In contemporary childhood environments, screen time has emerged as a significant lifestyle factor that may further influence this relationship [27]. Increased exposure to electronic devices, including televisions, tablets, smartphones, and video games, has been associated with delayed sleep onset, shorter sleep duration, and poorer sleep quality, particularly when device use occurs during evening hours [28]. Excessive screen use has also been linked to greater emotional difficulties, including increased irritability, reduced attentional control, and behavioral problems [29]. Additionally, screen-based behaviors contribute to sedentary lifestyles, potentially displacing time spent in physical activity. As such, screen time represents a relevant contextual variable that may influence the complex interplay between sleep, physical activity, and emotional regulation [30].

Socioeconomic and family contextual factors also play a significant role in shaping children’s sleep and emotional outcomes. Socioeconomic disadvantage has been associated with increased stress exposure, irregular daily routines, environmental noise, and limited access to recreational opportunities, all of which may negatively affect sleep quality and emotional development [31]. Parental employment status, as an indicator of socioeconomic position, may influence household routines, parental availability, stress levels, and opportunities for structured physical activity. Consequently, socioeconomic conditions may confound the relationship between sleep quality, physical activity, and emotional regulation [32].

Although previous research has examined the associations between sleep and emotional functioning, and between physical activity and psychological well-being, fewer studies have simultaneously explored these variables within the same model in elementary school children. In particular, existing research has rarely examined the relative contribution of sleep problems and habitual physical activity to children’s emotional functioning while simultaneously accounting for relevant contextual factors such as screen time and socioeconomic conditions. Moreover, evidence focusing specifically on middle childhood remains comparatively limited despite the important developmental changes occurring during this stage. Understanding the interrelationships among sleep quality, emotional regulation, and habitual physical activity during this developmental period while controlling for key sociodemographic and lifestyle variables may therefore provide important insights for preventive interventions aimed at promoting child mental health and well-being [33,34].

Therefore, the present study aimed to examine the relationship between sleep problems and emotional regulation in elementary school children, considering habitual physical activity levels and relevant sociodemographic and lifestyle confounding variables. We hypothesized that poorer sleep problems would be associated with greater emotional lability and lower emotional regulation, and that higher levels of physical activity would be associated with more adaptive emotional functioning. Additionally, we expected that screen time and socioeconomic indicators would influence these associations.

## 2. Materials and Methods

### 2.1. Study Design and Participants

A cross-sectional study was conducted among primary school children from Andalusia (Spain). All students enrolled in the upper primary grades of the participating school were invited to take part in the study. Of the invited students, 386 provided parental consent and completed the study assessments, constituting the final analytical sample. The school agreed to collaborate through an institutional agreement, and families were provided with detailed written information regarding the aims, procedures, and voluntary nature of the study prior to participation. The research protocol complied with the ethical standards established in the Declaration of Helsinki and received approval from the Ethics Committee of Midl-Atlantic University. Written informed consent was obtained from parents or legal guardians before data collection began.

The sample was drawn from a public school in Andalusia (Spain) and included students enrolled in upper primary grades. The school was situated in an urban area and served children from a mixed socioeconomic background typical of public educational settings in the region. Children were eligible to participate if they met the following inclusion criteria: (i) being between 9 and 12 years of age; (ii) absence of any medical condition that could contraindicate participation in the study procedures; (iii) no diagnosed intellectual disability that might interfere with comprehension of the questionnaires or compliance with the study protocol; and (iv) provision of written informed consent by a parent or legal guardian prior to participation.

### 2.2. Measures

#### 2.2.1. Emotional Regulation

Children’s emotional regulation was assessed using the Emotion Regulation Checklist (ERC) developed by Shields and Cicchetti [35]. The ERC is a caregiver-report instrument designed to evaluate emotion-related behaviors in everyday contexts. It is typically completed by parents, legal guardians, or teachers who have regular contact with the child and are therefore able to provide reliable observations of the child’s typical emotional functioning. The questionnaire consists of 24 items organized into two primary dimensions. The Emotion Regulation subscale assesses adaptive emotional functioning, including appropriate emotional expression, empathy, and the presence of positive affect. The Lability/Negativity subscale evaluates emotional instability and dysregulation, including mood variability, impulsivity, irritability, and disproportionate negative emotional responses. Items are rated on a 4-point Likert-type scale ranging from 1 (“rarely or never”) to 4 (“almost always”). Subscale scores are computed by summing the relevant items. Higher scores on the Emotion Regulation subscale reflect greater adaptive emotional control, whereas higher scores on the Lability/Negativity subscale indicate greater emotional dysregulation and difficulty managing affective responses. In the present study, the internal consistency of the ERC subscales was acceptable (Cronbach’s α = 0.725).

#### 2.2.2. Habitual Physical Activity

Habitual physical activity was assessed using the Physical Activity Questionnaire for Children (PAQ-C), a widely used and validated self-report instrument for school-aged children [36]. The PAQ-C provides a general estimate of moderate-to-vigorous physical activity performed during the previous seven days, capturing typical activity patterns within the child’s daily environment. The questionnaire is designed for group administration in school settings and requires approximately 10–15 min to complete. It includes several items that assess participation in physical activities across different contexts and time periods, such as leisure-time activity, physical education classes, recess, after-school periods, weekend activity, and overall perceived activity level. Each item is rated on a 5-point Likert-type scale, with higher scores reflecting greater levels of physical activity. A composite activity score is calculated by averaging the item responses, resulting in a continuous index ranging from 1 (low activity level) to 5 (high activity level). In this study, the Spanish-validated version was used [37]. In this study, the PAQ-C demonstrated good internal consistency (Cronbach’s α = 0.802).

#### 2.2.3. Sleep Problems

Sleep problems were evaluated using the Children’s Sleep Habits Questionnaire (CSHQ) [38], employing its validated Spanish version [39]. The CSHQ is a standardized and widely utilized instrument designed to assess sleep behaviors and common sleep difficulties in school-aged children. The questionnaire is completed by parents or legal guardians and gathers retrospective information regarding the child’s sleep patterns during a typical recent week. It comprises 33 items assessing the frequency of various sleep-related behaviors and disturbances. Each item is rated on a three-point Likert scale reflecting weekly occurrence: “rarely” (0–1 times per week), “sometimes” (2–4 times per week), and “usually” (5–7 times per week). The CSHQ yields both a total sleep disturbance score and eight domain-specific scores: bedtime resistance, sleep onset delay, sleep duration, sleep-related anxiety, night awakenings, parasomnias, sleep-disordered breathing, and daytime sleepiness. Domain and total scores are calculated by summing the relevant items, after reverse-coding positively worded items. Higher scores indicate a greater presence of sleep problems. Therefore, higher total CSHQ scores in this study were interpreted as reflecting poorer sleep problems. Although the CSHQ provides detailed information across eight subdomains, the present study used exclusively the total score as a global indicator of sleep problems. This approach was chosen to obtain a global measure of sleep problems and to maintain a parsimonious analytical model focused on overall sleep problems rather than domain-specific sleep behaviors. This methodological decision is consistent with numerous cross-sectional and observational studies in school-aged populations that have relied on the total CSHQ score as a summary index of sleep problems when the objective is to examine overall sleep problems rather than specific sleep disorders [40]. Furthermore, the use of a global score allows for a more parsimonious and statistically robust analytical approach in models including multiple psychological and behavioral variables, reducing the risk of type I error associated with testing multiple subscales simultaneously. In the present sample, the CSHQ total score showed acceptable internal consistency (Cronbach’s α = 0.788).

#### 2.2.4. Confounders

Age was considered a potential confounding variable given that neurocognitive and socioemotional development undergo substantial changes throughout middle childhood. Progressive maturation of brain structures, particularly those involved in executive functioning, directly influences children’s capacity for emotional regulation [41]. In parallel, sleep patterns evolve developmentally during this period, with changes observed in sleep duration, architecture, and circadian regulation [42,43]. Similarly, levels of physical activity tend to vary according to age and developmental stage [44,45]. For these reasons, age was included as a covariate in the analyses.

Socioeconomic status (SES) was also examined as a potential confounder. SES was estimated using parent-reported educational level and occupational status, following the classification criteria proposed by the Spanish Society of Epidemiology [46]. Although the original scale categorizes socioeconomic position into five levels, these were collapsed into three broader categories for analytical purposes due to the limited number of participants in the extreme groups: lower/lower-middle, middle, and upper-middle/upper. For the regression analyses, SES was coded as an ordinal variable with higher values representing higher socioeconomic status. Previous research has shown that socioeconomic disadvantage is associated with a higher prevalence of sleep disturbances in children [47,48].

Screen time was included as an additional confounding variable due to its documented associations with sleep, emotional functioning, and physical activity. Greater exposure to screen-based media has been linked to shorter sleep duration and poorer sleep problems, particularly when device use occurs during evening hours [28]. Excessive screen use has also been associated with increased emotional and behavioral difficulties [49]. Furthermore, screen-based behaviors contribute to sedentary lifestyles and may displace time otherwise devoted to physical activity [50]. Screen time was assessed using a parent-reported questionnaire adapted from previous research conducted in Spain [51]. Parents reported the duration of their child’s leisure-time use of electronic devices, including television, computers, tablets, smartphones, and video games, separately for weekdays and weekends. Reported weekly frequency and duration (in hours) were averaged and divided by seven to estimate mean daily screen exposure. In accordance with established guidelines, daily screen time was categorized as ≤1 h per day or >1 h per day for analytical purposes. This threshold was selected based on international recommendations suggesting that recreational screen exposure should be limited in school-aged children to minimize potential negative effects on sleep, emotional functioning, and physical activity behaviors. Based on this evidence, screen time was included as a covariate in the regression analyses to control for its potential confounding influence on the relationships between sleep problems, physical activity, and emotional regulation.

### 2.3. Procedure

Prior to data collection, school principals and teaching staff were formally contacted and informed about the aims, methodology, and practical requirements of the study. After institutional authorization was obtained, detailed information sheets were distributed to families describing the purpose of the research, the procedures involved, and the voluntary nature of participation. Written informed consent was secured from parents or legal guardians before any assessment was conducted. Both students and their families received clear explanations, provided in written and verbal form, regarding the study objectives and participation conditions. To ensure confidentiality and anonymity, each participant was assigned a unique identification code. All collected information was processed and stored in accordance with current data protection regulations.

Data collection took place during regular school hours on previously scheduled sessions specifically allocated for the study. Subsequently, children completed the self-report questionnaires in a structured classroom setting under researcher supervision to guarantee comprehension of the items and to minimize potential response bias.

The caregiver report questionnaires (Emotion Regulation Checklist and Infant Sleep Habits Questionnaire) were completed by the child’s primary caregiver, usually the mother or father, during scheduled sessions at school. Parents attended school to complete the questionnaires following standardized written instructions provided by the research team. Research staff were present to clarify any questions, ensuring consistency in the administration procedure. The research team reviewed the completed questionnaires and identified cases with substantial missing information.

### 2.4. Statistical Analysis

Data analyses were conducted using SPSS software (Version 22.0; IBM Corp., Armonk, NY, USA). Prior to hypothesis testing, the dataset was screened for accuracy, missing values, and extreme observations. No missing data were observed in the variables included in the analyses; therefore, no imputation procedures were required. Potential multivariate outliers were examined using Mahalanobis distance with a conservative significance threshold (*p* < 0.001). Univariate distributions were inspected through boxplots and standardized z-scores. No extreme cases were identified that required exclusion. Descriptive statistics were computed for all study variables. Continuous variables are reported as means and standard deviations, whereas categorical variables are presented as frequencies and percentages. Normality assumptions were evaluated using the Kolmogorov–Smirnov test, complemented by examination of skewness and kurtosis indices. Although formal tests indicated deviations from normality (*p* < 0.05), skewness and kurtosis values remained within acceptable limits (±1.5), supporting the use of parametric procedures. Homogeneity of variances between boys and girls was assessed using Levene’s test. Sex differences in primary variables (CSHQ total score, ERC subscales, and PAQ-C score) were analyzed using independent-samples *t*-tests. Bivariate associations among sleep problems, emotional regulation dimensions, and physical activity were explored using Pearson correlation coefficients. To investigate the relationship between sleep problems and emotional regulation while accounting for potential confounders, multiple linear regression analyses were performed using the enter method. Separate models were estimated for each ERC subscale (Emotion Regulation and Lability/Negativity) as dependent variables. The CSHQ total score and PAQ-C score were entered as primary predictors. Age, sex, socioeconomic status, and daily screen time were included as covariates in all models. Because the PAQ-C and CSHQ scores showed a strong correlation, separate regression models were estimated for each predictor to minimize potential multicollinearity effects and to examine their independent associations with emotional outcomes. Multicollinearity diagnostics were examined through variance inflation factors (VIF). Standardized regression coefficients (β) and corresponding significance levels were reported. Statistical significance was established at *p* < 0.05.

## 3. Results

Descriptive statistics for the main study variables are presented in Table 1. The final sample consisted of 386 elementary school children aged between 9 and 12 years (mean age = 11.15 years, SD = 0.66). Both boys and girls were included in the study, allowing the examination of potential sex differences in the analyzed variables. Of the total participants, 176 were boys (45.6%) and 210 were girls (54.4%). The mean age of participants was 11.15 ± 0.66 years. The average CSHQ total score was 69.67 ± 16.58, indicating the overall level of sleep-related problems in the sample. Higher scores reflect greater sleep disturbances and therefore poorer sleep problems. Regarding emotional functioning, the mean score for the Emotion Regulation subscale was 37.49 ± 2.16, whereas the mean Lability/Negativity score was 24.96 ± 2.39. The average PAQ-C score was 3.35 ± 0.42, reflecting a moderate level of habitual physical activity. Normality assumptions were evaluated using the Kolmogorov–Smirnov test. Although the formal tests indicated statistically significant deviations from normality (*p* < 0.05), skewness and kurtosis values for all primary variables were within acceptable limits (±1.5). Given the large sample size (*n* = 386) and the approximate symmetry of the distributions, parametric analyses were considered appropriate for subsequent statistical procedures. No extreme univariate or multivariate outliers were identified that required exclusion from the analyses.

### 3.1. Associations Among Sleep Problems, Emotional Regulation, and Physical Activity

Significant associations were observed among sleep problems, emotional regulation, and habitual physical activity (Table 2). Sleep problems (CSHQ total score) were strongly and negatively associated with the Emotion Regulation subscale (r = −0.745, *p* < 0.001), indicating that higher levels of sleep disturbances were linked to poorer adaptive emotional regulation. Conversely, sleep problems were positively associated with Lability/Negativity (r = 0.510, *p* < 0.001), suggesting that children experiencing greater sleep difficulties exhibited increased emotional instability. Habitual physical activity (PAQ-C score) showed a positive association with Emotion Regulation (r = 0.551, *p* < 0.001) and a negative association with Lability/Negativity (r = −0.406, *p* < 0.001). In addition, PAQ-C scores were strongly and inversely related to sleep problems (r = −0.771, *p* < 0.001), indicating that higher physical activity levels were associated with fewer sleep-related difficulties. Age was not significantly associated with the primary study variables (*p* > 0.05).

### 3.2. Multiple Linear Regression Analysis Between Emotional Regulation, Sleep Problems, and Physical Activity

Two separate multiple linear regression models were conducted to examine the independent associations of sleep problems and habitual physical activity with emotional regulation, controlling for age, sex, socioeconomic status, and daily screen time (Table 3). In the first model, sleep problems (CSHQ total score) were entered as the main predictor. The overall model was statistically significant, F(5, 380) = 95.49, *p* < 0.001, accounting for 55.1% of the variance in emotional regulation (adjusted R^2^ = 0.551). Sleep problems were a strong and significant negative predictor of emotional regulation (β = −0.663, *p* < 0.001), indicating that higher levels of sleep disturbances were associated with lower adaptive emotional regulation, independent of sociodemographic and lifestyle factors. None of the covariates included in the model (age, sex, socioeconomic status, or screen time) reached statistical significance (all *p* > 0.05). In the second model, habitual physical activity (PAQ-C score) was examined as the primary predictor. This model was also statistically significant, F(5, 380) = 81.95, *p* < 0.001, explaining 51.2% of the variance in emotional regulation (adjusted R^2^ = 0.512). However, physical activity was not significantly associated with emotional regulation after adjustment for covariates (β = 0.057, *p* = 0.280). In contrast, socioeconomic status (β = −0.0453, *p* < 0.001) (indicating lower emotional regulation scores among children from lower socioeconomic backgrounds) and screen time (β = −0.265, *p* < 0.001) were significantly associated with emotional regulation in this model. Overall, sleep problems demonstrated a stronger association with emotional regulation compared to habitual physical activity. However, these findings should be interpreted with caution because physical activity and sleep problems were not included simultaneously in the same regression model due to their strong intercorrelation. Therefore, conclusions regarding the relative contribution of each predictor should be considered indicative rather than definitive. While both models explained a substantial proportion of variance, only sleep problems remained a significant independent predictor when accounting for relevant sociodemographic and lifestyle factors. Specifically, tolerance and VIF values for sleep problems were 0.078 and 12.82, respectively, while the corresponding values for physical activity were 0.400 and 2.50. The remaining covariates showed acceptable multicollinearity levels (VIF < 8).

To examine the relative contribution of sleep problems and physical activity, an additional regression model including both predictors simultaneously was conducted. Sleep problems remained significantly associated with emotional regulation (β = −0.710, *p* < 0.001), whereas physical activity was not statistically significant (β = −0.057, *p* = 0.295). Multicollinearity diagnostics indicated elevated variance inflation factors for sleep problems (VIF = 12.82), reflecting the strong correlation between sleep and physical activity observed in the dataset. Given the high multicollinearity observed between sleep problems and physical activity, hierarchical regression models comparing the incremental variance explained by each predictor were not considered appropriate, as the shared variance between these variables could distort the interpretation of ΔR^2^ values.

### 3.3. Multiple Linear Regression Analysis Between Lability/Negativity, Sleep Problems, and Physical Activity

A multiple linear regression analysis was conducted to examine whether sleep problems predicted Lability/Negativity after controlling for age, sex, socioeconomic status, and daily screen time (Table 4). The overall model was statistically significant, F(5, 380) = 28.09, *p* < 0.001, explaining 27.0% of the variance in Lability/Negativity (R^2^ = 0.270; adjusted R^2^ = 0.260). Sleep problems emerged as a significant positive predictor of Lability/Negativity (β = 0.752, *p* < 0.001), indicating that higher levels of sleep disturbances were associated with greater emotional instability. None of the covariates (age, sex, socioeconomic status, or screen time) reached statistical significance in this model (*p* > 0.05). Collinearity diagnostics indicated acceptable tolerance values, although sleep showed elevated VIF values, consistent with its strong association with emotional variables. A second regression model was conducted replacing sleep problems with physical activity (PAQ-C score) as the primary predictor, while maintaining the same covariates. The overall model was also statistically significant, F(5, 380) = 22.39, *p* < 0.001, accounting for 22.8% of the variance in Lability/Negativity (R^2^ = 0.228; adjusted R^2^ = 0.217). Physical activity was a significant negative predictor of Lability/Negativity (β = −0.136, *p* = 0.041), indicating that higher levels of habitual physical activity were associated with lower emotional instability. Additionally, socioeconomic status emerged as a significant positive predictor (β = 0.277, *p* = 0.001), suggesting that lower socioeconomic conditions were associated with higher emotional lability. Age, sex, and screen time were not significant predictors (*p* > 0.05). Collinearity statistics were within acceptable ranges (VIF < 5), supporting the adequacy of the regression estimates.

To examine the relative contribution of sleep problems and physical activity, additional regression models including both predictors simultaneously were conducted. For emotional regulation, sleep problems remained significantly associated with the outcome (β = −0.710, *p* < 0.001), whereas physical activity was not statistically significant (β = −0.057, *p* = 0.295). A similar pattern was observed for emotional lability, with sleep problems showing a significant positive association (β = 0.737, *p* < 0.001), while physical activity was not significant (β = −0.018, *p* = 0.793). Multicollinearity diagnostics indicated elevated variance inflation factors for sleep problems (VIF = 12.82), reflecting the strong correlation between sleep and physical activity observed in the dataset.

## 4. Discussion

The present study aimed to examine the associations between sleep problems, emotional regulation, and habitual physical activity in elementary school children, while controlling for relevant sociodemographic and lifestyle factors. The findings indicate that sleep problems emerged as the most robust and consistent association with emotional functioning. Specifically, greater sleep problems were strongly associated with lower adaptive emotional regulation and higher emotional lability. Although physical activity showed significant bivariate associations with both emotional dimensions, its predictive value weakened after adjustment for covariates, particularly in the case of adaptive emotional regulation.

The strong association observed between sleep problems and both emotional regulation and lability is consistent with a growing body of research emphasizing the central role of sleep-in children’s socioemotional development. Previous studies have shown that insufficient or poor-quality sleep is associated with increased irritability, emotional reactivity, internalizing symptoms, and behavioral dysregulation in school-aged children [42,47]. Similarly, longitudinal evidence suggests that sleep difficulties in childhood predict later emotional and behavioral problems [52]. Our findings extend this literature by demonstrating that sleep problems remained significantly associated with emotional functioning even after controlling for socioeconomic status, screen time, and physical activity levels. From a neurodevelopmental perspective, these associations may be explained by the impact of sleep on prefrontal cortical maturation and its regulatory influence over limbic structures such as the amygdala, which are central to emotional modulation [53]. Although the present findings highlight a strong association between sleep problems and emotional functioning, the cross-sectional design of the study does not allow causal direction to be established. It is plausible that the relationship between sleep and emotional regulation is bidirectional. For example, children experiencing emotional dysregulation, stress, or heightened emotional reactivity may also develop sleep difficulties, including problems with sleep onset or nighttime awakenings. Conversely, insufficient or disturbed sleep may impair prefrontal regulatory processes and increase emotional reactivity. Future longitudinal and experimental studies are therefore needed to clarify the temporal dynamics and potential bidirectional mechanisms linking sleep and emotional functioning in childhood. The relatively high correlations observed between sleep problems and emotional functioning variables in Table 2 also warrant methodological consideration. First, some degree of construct overlap may exist between sleep disturbances and emotional dysregulation, as sleep difficulties in children may manifest behaviorally through irritability, mood instability, or increased emotional reactivity. Second, both sleep problems and emotional regulation were assessed using caregiver-reported questionnaires, which may introduce common method variance and potentially inflate the magnitude of the observed associations. Finally, the strong correlation observed between sleep problems and physical activity suggests the possibility of multicollinearity when these predictors are considered simultaneously in regression models. These methodological factors should therefore be taken into account when interpreting the magnitude of the reported relationships.

Interestingly, while habitual physical activity was positively associated with emotional regulation and negatively associated with lability at the correlational level, its predictive strength diminished in adjusted models. This partially contrasts with previous findings in adolescent populations showing consistent positive associations between moderate-to-vigorous physical activity and emotional competence [54,55]. However, several studies have reported more modest or context-dependent effects in younger children, suggesting that the relationship between physical activity and emotional outcomes may be mediated by factors such as sleep problems, social context of activity, or program structure [56,57]. In line with this interpretation, our results showed that physical activity retained a modest association with lower emotional lability but did not independently predict adaptive emotional regulation when sleep was considered. This suggests that sleep may represent an important factor associated with emotional functioning during middle childhood.

The differential findings between emotional regulation and lability/negativity also merit consideration. Sleep problems explained a substantial proportion of variance in both dimensions, whereas physical activity showed a weaker and more specific association with emotional instability. This pattern aligns with theoretical models proposing that emotional lability reflects heightened affective reactivity, which may be particularly sensitive to physiological dysregulation associated with sleep deprivation [58]. In contrast, adaptive emotional regulation involves executive functions and cognitive control processes that may require sustained developmental maturation and environmental scaffolding [59]. Therefore, while physical activity may contribute indirectly through improvements in mood and stress reduction, sleep may play an important role in the neurocognitive mechanisms underlying emotional control.

Socioeconomic status emerged as a significant predictor in some models, particularly in relation to emotional lability. This finding is consistent with research indicating that socioeconomic disadvantage is associated with higher exposure to stressors, inconsistent routines, and environmental conditions that may negatively affect both sleep and emotional stability [60,61]. These contextual influences highlight the importance of considering family and environmental factors when examining behavioral determinants of emotional health. Screen time, although correlated with emotional variables at the bivariate level, did not consistently predict outcomes in adjusted models, which may suggest that its impact operates primarily through sleep disruption rather than directly influencing emotional processes.

Taken together, the present findings support the growing recognition of sleep as a critical component of the 24 h movement behavior framework. While physical activity remains essential for children’s physical and psychological health, the results of this study suggest that sleep problems may be closely associated with emotional functioning during middle childhood. From a practical perspective, promoting healthy sleep habits could represent a promising direction for future school-based well-being initiatives. Educational strategies aimed at increasing awareness of sleep hygiene may also be beneficial, particularly when integrated with programs that encourage regular physical activity. However, longitudinal and intervention studies are needed to determine whether improving sleep behaviors leads to measurable improvements in children’s emotional regulation.

Several limitations should be acknowledged. The cross-sectional design precludes causal inference, and longitudinal studies are needed to clarify temporal directionality. The reliance on questionnaire-based measures may introduce reporting bias. The reliance on questionnaire-based measures to assess lifestyle behaviors (sleep problems, physical activity, and emotional regulation) may introduce reporting bias and may not capture behavioral patterns with the same level of precision as objective methods such as actigraphy or accelerometry. Although the instruments used in this study have been widely employed and validated in school-aged children, indirect assessments may influence the strength of the observed associations. Furthermore, although the chronological age range of participants was relatively narrow (9–12 years), the study did not include measures of biological maturation or pubertal status. Differences in biological development during late childhood may influence sleep patterns, emotional regulation, and physical activity behaviors, which could not be accounted for in the present analyses. Additionally, the sample was drawn from a single public school located in an urban area of southern Spain, which may limit generalizability. School-level characteristics such as institutional routines, educational practices, and local socioeconomic context may influence children’s lifestyle behaviors and emotional functioning. Therefore, the results should be interpreted with caution when extrapolating to other educational contexts. Future research should incorporate objective sleep and activity measures (e.g., actigraphy), consider indicators of biological maturation, differentiate between types of physical activity (team-based vs. individual), and explore potential mediational pathways linking physical activity, sleep, and emotional outcomes. Despite these limitations, this study contributes novel evidence by simultaneously examining sleep problems, physical activity, and emotional regulation in a sample of elementary school children while controlling for key contextual variables. By demonstrating the stronger and more consistent role of sleep, the findings underscore the need to integrate sleep promotion strategies into child mental health and school-based well-being programs.

## 5. Conclusions

The present study identified significant associations between sleep problems, emotional regulation, and emotional lability in a sample of elementary school children. Specifically, sleep problems were strongly associated with lower adaptive emotional regulation and higher emotional instability after accounting for relevant sociodemographic and lifestyle factors. Although habitual physical activity was related to emotional outcomes at the bivariate level, these associations were less consistent in adjusted models. Additional analyses including both sleep problems and physical activity simultaneously suggested that sleep problems remained significantly associated with emotional outcomes, whereas physical activity did not reach statistical significance. However, given the strong correlation observed between these variables and the cross-sectional nature of the study, the relative contribution of these lifestyle factors should be interpreted with caution. Overall, these findings highlight the potential relevance of sleep problems as a factor associated with children’s emotional functioning during middle childhood. From a practical perspective, promoting healthy sleep habits may represent a promising direction for future research and school-based well-being initiatives. Nevertheless, longitudinal and experimental studies are needed to clarify the temporal direction of these relationships and to determine whether changes in sleep behaviors may contribute to improvements in emotional regulation over time.

## Figures and Tables

**Table 1:** Sociodemographic and sleep-related characteristics of the sample by sex.

Variable		TOTAL (N = 386)	Boys (N = 176)	Girls (N = 210)	*p* Value
Age (years)		11.15 ± 0.66	11.20 ± 0.64	11.10 ± 0.67	0.278
Screen time	Screen time (≤1 h/day)	194 (50.3%)	83 (21.5%)	111 (28.8%)	0.665
	Screen time (>1 h/day)	192 (49.7%)	93 (24.1%)	99 (25.6%)	
Socioeconomic status	Lower-Upper lower	110 (28.5%)	43 (11.1%)	67 (17.4%)	0.141
	Lower-Middle	157 (40.7%)	71 (18.4%)	86 (22.3%)	
	Upper middle-Upper	119 (30.8%)	62 (16.1%)	57 (14.8%)	

**Table 2:** Pearson Correlations Among Study Variables.

Variable	1	2	3	4	5
Emotion Regulation	—				
Lability/Negativity	−0.388 ***	—			
Sleep Problems (CSHQ total)	−0.745 ***	0.510 ***	—		
Physical Activity (PAQ-C)	0.551 ***	−0.406 ***	−0.771 ***	—	
Age	−0.004	0.038	0.010	−0.069	—

Note. 1 = Emotion Regulation; 2 = Lability/Negativity; 3 = Sleep Problems (CSHQ total score); 4 = Physical Activity (PAQ-C score); 5 = Age. Values represent Pearson correlation coefficients (two-tailed). *** *p* < 0.001.

**Table 3:** Multiple linear regression models predicting emotional regulation.

Variable	Model 1 (Sleep Problems)					Model 2 (Physical Activity)				
	B	β	*p*	95% CI (Lower)	95% CI (Upper)	B	β	*p*	95% CI (Lower)	95% CI (Upper)
Sleep problems (CSHQ total)	−0.088	−0.663	<0.001	−0.118	−0.058	—	—	—		
Physical activity (PAQ-C)	—	—	—			0.276	0.057	0.280	−0.791	0.241
Age	0.005	0.001	0.966	−0.221	0.231	−0.026	−0.008	0.831	−0.232	0.221
Sex	0.072	0.016	0.640	−0.230	0.374	−0.047	−0.011	0.769	−0.220	0.384
Socioeconomic status	−0.076	−0.027	0.780	−0.612	0.460	−1.295	−0.453	<0.001	−0.610	0.462
Screen time	−0.292	−0.066	0.307	−0.854	0.269	−1.167	−0.265	<0.001	−0.842	0.282
Model fit										
F (df)	F(5, 380) = 95.49		<0.001			F(5, 380) = 81.95		<0.001		
R^2^	0.557					0.551				
Adjusted R^2^	0.551					0.512				
N	386					286				

Note. B = unstandardized regression coefficient; β = standardized regression coefficient; CI = confidence interval; N = number of observations. Model 1 included sleep problems (CSHQ total score) as the primary predictor; Model 2 included physical activity (PAQ-C score) as the primary predictor. All models were adjusted for age, sex, socioeconomic status, and daily screen time.

**Table 4:** Multiple linear regression models predicting lability/negativity.

Variable	Model 1 (Sleep Problems)					Model 2 (Physical Activity)				
	B	β	*p*	95% CI (Lower)	95% CI (Upper)	B	β	*p*	95% CI (Lower)	95% CI (Upper)
Sleep problems (CSHQ total)	0.148	0.752	<0.001	0.084	0.206	—	—	—		
Physical activity (PAQ-C)	—	—	—			−0.978	−0.136	0.041	−1.112	0.850
Age	0.110	0.022	0.613	0.110	0.136	0.136	0.028	0.545	−0.26	0.536
Sex	−0.385	−0.059	0.187	−0.956	0.195	−0.183	−0.028	0.540	−0.956	0.195
Socioeconomic status	−0.708	−0.167	0.173	−1.727	0.313	1.171	0.277	0.001	−1.727	0.313
Screen time	−0.687	−0.105	0.206	−1.751	0.388	0.683	0.105	0.149	−1.751	0.388
Model fit										
F (df)	F(5, 380) = 28.09		<0.001			F(5, 380) = 22.39		<0.001		
R^2^	0.270					0.270				
Adjusted R^2^	0.260					0.258				
N	386					386				

Note. B = unstandardized regression coefficient; β = standardized regression coefficient; CI = confidence interval; N = number of observations. Model 1 included sleep problems (CSHQ total score) as the primary predictor; Model 2 included physical activity (PAQ-C score) as the primary predictor. All models were adjusted for age, sex, socioeconomic status, and daily screen time.

## Data Availability

The data presented in this study are available on request from the corresponding author. The datasets generated and analyzed during the current study include sensitive information collected from minors, which requires strict confidentiality and protection in accordance with ethical and legal regulations. The data are not publicly available because, due to the sensitive nature of the questions asked in this study, participants and their legal guardians were assured that the raw data would remain confidential and would not be publicly shared. Furthermore, the participants constitute a vulnerable population as they are minors, and data handling procedures were designed to ensure maximum protection of their privacy and personal information in line with ethical approval requirements and data protection legislation.

## Abbreviations

The following abbreviations are used in this manuscript:

CSHQ	Children’s Sleep Habits Questionnaire
ERC	Emotion Regulation Checklist
PAQ-C	Physical Activity Questionnaire for Children
MVPA	Moderate-to-Vigorous Physical Activity
SES	Socioeconomic Status
